# Peer-led recovery groups for people with psychosis in South Africa (PRIZE): Results of a randomized controlled feasibility trial

**DOI:** 10.1017/S2045796024000556

**Published:** 2024-10-11

**Authors:** Laura Asher, Bongwekazi Rapiya, Julie Repper, Tarylee Reddy, Bronwyn Myers, Gill Faris, Inge Petersen, Charlotte Hanlon, Carrie Brooke-Sumner

**Affiliations:** 1 Unit of Lifespan and Population Health, Nottingham Centre of Public Health and Epidemiology, School of Medicine, University of Nottingham, Nottingham, UK; 2 Institute of Mental Health, University of Nottingham, Nottingham, UK; 3 Mental Health, Alcohol, Substance Use and Tobacco Research Unit, South African Medical Research Council, Francie Van Zijl Drive, Parow Valley, Cape Town, South Africa; 4 Implementing Recovery through Organisational Change, Nottingham, UK; 5 Biostatistics Research Unit, South African Medical Research Council, Durban, South Africa; 6 Curtin enAble Institute, Curtin University, WA, Australia; 7 Department of Psychiatry and Mental Health, University of Cape Town, J-Block, Groote Schuur Hospital, Observatory, Cape Town, South Africa; 8 Purposeful People Development, Cape Town, South Africa; 9 Centre for Rural Health, College of Health Sciences, University of KwaZulu-Natal, Durban, South Africa; 10 Centre for Global Mental Health, Health Service and Population Research Department, Institute of Psychiatry, Psychology and Neuroscience, King’s College London, London, UK; 11 Addis Ababa University, College of Health Sciences, School of Medicine, Department of Psychiatry, Addis Ababa, Ethiopia; 12 Alan J. Flisher Centre for Public Mental Health, Department of Psychiatry & Mental Health, University of Cape Town, Cape Town, South Africa

**Keywords:** community mental healthcare, peer support, personal recovery, Psychosis, psychosocial intervention

## Abstract

**Aims:**

The aims of this feasibility trial were to assess the acceptability and feasibility of peer-led recovery groups for people with psychosis in a low-resource South African setting, to assess the feasibility of trial methods, and to determine key parameters in preparation for a definitive trial.

**Methods:**

The design was an individually randomised feasibility trial comparing recovery groups in addition to treatment as usual (TAU) with TAU alone. Ninety-two isiXhosa-speaking people with psychosis and forty-seven linked caregivers were recruited from primary care clinics and randomly allocated to trial arms in a 1:1 allocation ratio. TAU comprised anti-psychotic medication delivered in primary care. The intervention arm comprised six recovery groups including service users and caregivers. Two-hour recovery group sessions were delivered weekly in a 2-month auxiliary social worker (ASW)-led phase, then a 3-month peer-led phase. To explore acceptability and feasibility, a mixed methods process evaluation included 25 in-depth interviews and 2 focus group discussions at 5 months with service users, caregivers and implementers, and quantitative data collection including attendance and facilitator competence. To explore potential effectiveness, quantitative outcome data (functioning, relapse, unmet needs, personal recovery, stigma, health service use, medication adherence and caregiver burden) were collected at baseline, 2 months and 5 months post randomisation. Trial registration: PACTR202202482587686.

**Results:**

Qualitative interviews revealed that recovery groups were broadly acceptable with most participants finding groups to be an enjoyable opportunity for social interaction, and joint problem-solving. Peer facilitation was a positive experience; however a minority of participants did not value expertise by lived experience to the same degree as expertise of professional facilitators. Attendance was moderate in the ASW-led phase (participants attended 59% sessions on average) and decreased in the peer-led phase (41% on average). Participants desired a greater focus on productive activities and financial security. Recovery groups appeared to positively impact on relapse. Relapse occurred in 1 (2.2%) of 46 participants in the recovery group arm compared to 8 (17.4%) of 46 participants in the control arm (risk difference -0.15 [95% CI: −0.26; −0.05]). Recovery groups also impacted on the number of days in the last month totally unable to work (mean 1.4 days recovery groups vs 7.7 days control; adjusted mean difference −6.3 [95%CI: −12.2; −0.3]). There were no effects on other outcomes.

**Conclusion:**

Peer-led recovery groups for people with psychosis in South Africa are potentially acceptable, feasible and effective. A larger trial, incorporating amendments such as increased support for peer facilitators, is needed to demonstrate intervention effectiveness definitively.

## Introduction

Globally, people with psychosis experience disability, social exclusion and economic hardship (Patel *et al.*, 2018). The importance of community-based psychosocial support in addressing these difficulties is supported by a growing evidence base in low- and middle-income countries (LMIC)(Asher *et al.*, 2022; Brooke-Sumner *et al.*, 2015), as well as being a strategic priority in the WHO Mental Health Action Plan (WHO, 2021a). Yet real world provision of psychosocial interventions remains largely absent. In South Africa, whilst in some areas people with psychosis have access to primary care clinic-based outpatient services (primarily free anti-psychotic medication), and inpatient care, community-based support is lacking. In South Africa 25% of service users are readmitted to hospital within three months of discharge, highlighting the insufficiency of community care (Docrat *et al.*, 2019). The 2018 Life Esidimeni tragedy, in which 144 service users discharged from inpatient care to non-governmental organisations died because of neglectful care is a further example (Freeman, 2018).

Feasible evidence-based approaches are urgently needed to address this shortfall. The WHO promotes peer support workers as a means of expanding coverage of community-based mental healthcare (WHO, 2021a). As a form of task-sharing, peer support may be an advantageous approach in settings like South Africa where there are few mental health professionals. Peer support is provided by people with lived experience of mental health conditions in group or individual formats and includes emotional support, advocacy and activities to promote social inclusion (WHO, 2021b). With peer support, there is a strong emphasis on personal recovery, that is the ‘deeply personal, unique process of changing ones’ attitude, values, feelings, goals, skills and/or roles’ (Anthony, 1993), through focusing on issues of importance to service users. Peer support may reduce self-stigmatisation and instil hope for recovery through mutual problem solving, positive role modelling and building self-confidence through meeting others with similar experiences (Bellamy *et al.*, 2017). Peer support groups may be particularly appropriate in LMIC settings where family and socially oriented mechanisms of recovery are prominent (Gamieldien *et al.*, 2021).

Despite a recent increase in evaluations of mental health peer support in LMIC (Le *et al.*, 2022; Nixdorf *et al.*, 2022), the vast majority of studies have so far been conducted in high-income countries (Chien *et al.*, 2019; Lyons *et al.*, 2021; White *et al.*, 2020). There is emerging evidence that group peer support interventions are effective in supporting personal recovery among people with schizophrenia (Lyons *et al.*, 2021). Yet there is an absence of high-quality evidence of the acceptability, feasibility and effectiveness of group-based peer support approaches for people with psychosis in LMIC (Kohrt *et al.*, 2018). This knowledge is needed to inform future investment in these kinds of services, particularly in settings such as South Africa where mental health resources are so constrained. To address this gap, we developed the peer-led recovery groups for people with psychosis in South Africa (PRIZE) intervention, building on our model of group psychosocial rehabilitation previously piloted in South Africa’s North West Province (Brooke-Sumner *et al.*, 2016, 2018). The PRIZE intervention was grounded in the priorities of service users and caregivers identified in our in-depth formative research, to be reported separately.

The primary objective of this randomised feasibility trial was to assess the acceptability and feasibility of peer-led recovery groups for people with psychosis in a low-resource South African setting. Secondary objectives were to assess the feasibility of trial methods, to determine key parameters in preparation for a definitive trial and to explore the potential effectiveness of recovery groups plus treatment as usual (TAU) compared to TAU alone.

## Methods

### Study design and setting

**Figure 1.**
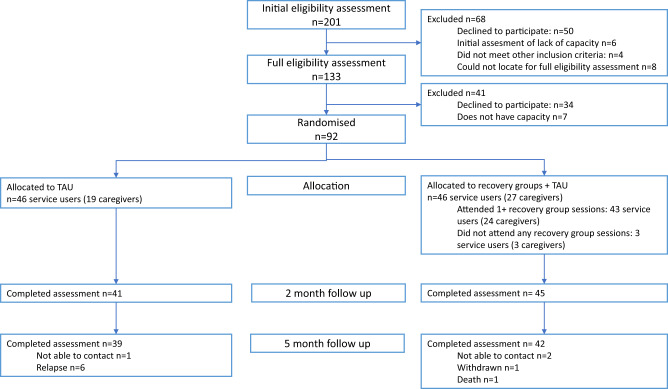
PRIZE feasibility trial flow chart.

The design was an individually randomised parallel group feasibility trial comparing recovery groups in addition to TAU compared to TAU alone in a 1:1 allocation ratio (Figure 1). A qualitative and quantitative process evaluation was used to address the primary objective to assess intervention acceptability and feasibility. Quantitative analysis of trial outcome data was used to assess the secondary objective to explore potential intervention effectiveness. The study was registered at the Pan-African Clinical Trials Register on 28 February 2022 (PACTR202202482587686) and the protocol is published (Asher *et al.*, 2023).

The study site was Nelson Mandela Bay Metropolitan district in the Eastern Cape province, which has the lowest gross domestic product per capita in South Africa. The district has eight primary care clinics providing mental healthcare for people with psychosis delivered by psychiatric nurses, including intermittently available free medication, but no psychosocial support.

### Recruitment and participants

Trial participants were service users and caregivers. Service user eligibility criteria were: (i) clinical diagnosis of psychosis, including schizophrenia, schizoaffective disorder or dual diagnosis with alcohol use disorder; (ii) ≥18 years old; (iii) spoke isiXhosa and (iv) had decision-making capacity to give informed consent to study participation. Caregiver eligibility criteria were: (i) primary caregiver for a participating service user; (ii) ≥18 years old and (iii) spoke isiXhosa. The recovery group facilitators and supervisor also participated in the process evaluation. Four individuals who met the eligibility criteria but who declined participation were invited to a qualitative interview.

We recruited participants at seven clinics in areas with high levels of economic and social adversity and which serve a predominantly Black African, isiXhosa-speaking population. Service users were recruited at clinics after their regular appointments, where an assessor completed an initial eligibility assessment. Diagnosis of psychosis was determined by the treating psychiatric nurse using clinical judgement. Service users were invited to identify a primary caregiver to participate in the study, but those without a caregiver were still eligible. Full eligibility and consent procedures, including capacity assessment, were then undertaken at a home visit by the trial social worker after providing detailed information about the study. Capacity to consent was assessed using a modified capacity assessment form shown to be feasible in other LMIC settings (Hanlon *et al.*, 2016; Mugisha *et al.*, 2017). Participants were provided with a R150 (USD 8) voucher at each assessment. Written informed consent was obtained for all participants. As this was a feasibility study it was not powered to determine effectiveness. We anticipated our target sample size of 100 service users would be sufficient to assess intervention acceptability and feasibility (Eldridge *et al.*, 2016).

### Interventions

The randomization code was generated by an independent statistician using permuted block randomisation. Randomisation was stratified by clinic catchment area. The recruiting trial social worker supplied the study coordinator with details of recruited participants. The study coordinator determined the allocation code using the Redcap randomisation module. The assessors were masked to allocation status.

#### Treatment as usual (TAU)

TAU consisted of treatment at the clinic, delivered mainly by psychiatric nurses. Monthly appointments are the norm. Treatment includes ongoing provision of anti-psychotic medication and symptom checking. Nurses can refer to a physician within the clinic, if available, or to inpatient care at local hospitals, for complex needs.

#### Recovery groups

**Figure 2.**
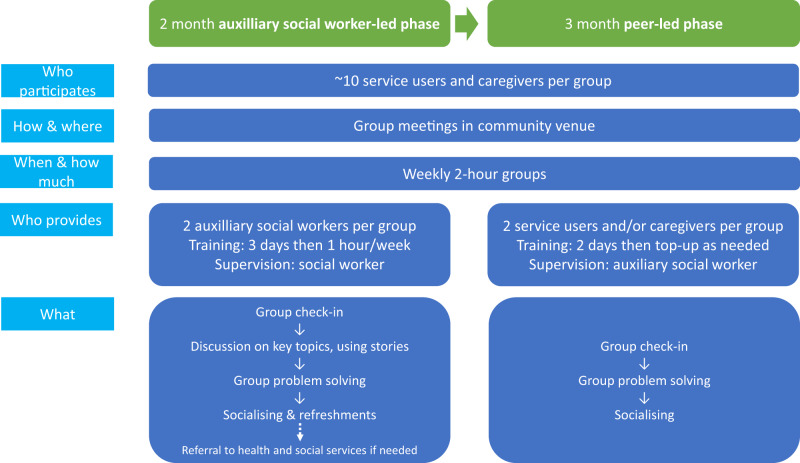
PRIZE recovery group model.

The intervention arm comprised six recovery groups, each linked to a clinic catchment area and including both service users and caregivers (see Figure 2). The PRIZE model is grounded in recovery-focused core values of building hope, opportunity and control. All group members were valued as experts by experience with knowledge and skills that formed the core of the group ‘content’ and value. Recovery groups were delivered in a 2-month auxiliary social worker (ASW)-facilitated phase, then a 3-month supported peer-led phase (Asher *et al.*, 2023). Indlela Mental Health (IMH) is a charitable organization mainly offering community-based psychosocial support for people with intellectual disabilities in the study district. Two female ASWs currently working at IMH, along with two female assistant facilitators, facilitated the recovery groups. Each pair facilitated three groups. Facilitators were initially trained for 3 days by an adult education specialist and the study coordinator, followed by 1 hour/week training staggered between group sessions, following the apprenticeship model of training (Murray *et al.*, 2011). Manualised training, using participatory methods, covered: recovery group values, facilitation skills, session content and supervision processes. Recovery group sessions were weekly, lasting 2 hours and held in community centres. The ASW-led phase comprised nine manualized sessions, covering recovery planning and other topics e.g., Building Self Esteem. Sessions included check-in, group problem solving; information provision; and informal socializing (see Supplementary File 1 for session outlines and https://www.mhinnovation.net/innovations/peer-led-recovery-groups-people-psychosis-south-africa-prize for manual). Group problem solving was encouraged to promote ownership and self-determination and enable sharing of coping strategies. Refreshments were provided for the ASW-led phase. ASWs were supervised by a social worker employed by IMH. Supervision was intended to comprise a weekly debrief and a monthly observed session, at which the social worker would complete an observational competency assessment (GroupACT) and provide feedback. The GroupAct tool assesses psychosocial group facilitation skills by scoring on unhelpful or potentially harmful behaviours, basic and advanced helping skills. The seven items include empathy, collaborative problem-solving and confidentiality (Pedersen *et al.*, 2021).

At week 4–5 of the ASW-facilitated phase, two peer facilitators (service users or caregivers) were identified from each group through self and group member nominations. Peer facilitator training was intended to happen over four half-day sessions. Peer facilitators who attended the first training felt uncomfortable attending a central venue. Training was reconfigured to be delivered by ASWs immediately before group meetings in the usual group venue, followed by on-the-job mentorship during group sessions. The peer-led phase was intended to comprise 13 sessions, covering check-in, group problem-solving and socializing. Refreshments were not provided to minimise costs and ensure that the intervention we evaluated was scalable in real world contexts with minimal resources. Peer facilitators were given a two-page illustration-based universal session outline in isiXhosa. It was intended that ASWs would observe the first two sessions, then attend monthly (including GroupACT assessment to identify training needs and give feedback). ASWs had weekly telephone debriefs with peer facilitators. Peer facilitators were not renumerated. To promote participation, ASWs contacted each participant by text/phone prior to each session. A reminder card was given for the following week’s session. ASWs contacted non-attending group members to encourage attendance.

### Measures

#### Process evaluation

To assess acceptability and feasibility, one or more process indicators spanning qualitative and quantitative data were selected for each precondition (intermediate outcome) on the theory of change (P1–P20 Supplementary File 2; Table 1). Four in-depth interviews (IDIs) with service users and caregivers declining to participate in the study were conducted at baseline to understand barriers to participation. Twenty-five IDIs were conducted at 5 months post-recruitment with service users, caregivers, ASWs, and the supervisor, to assess the acceptability and feasibility of peer-led groups. Two focus group discussions (FGDs) were held with peer facilitators to explore adequacy of training and self-perception of facilitation skills. IDIs and FGDs were conducted in isiXhosa and were audio-recorded. Quantitative data were collected to quantify training, supervision and session attendance, peer facilitators identified, session reminders attempted and conveyed, referrals by ASWs, and peer-led session shadowing by ASWs. Group facilitation skills of ASW and peer facilitators were assessed by the study coordinator with the GroupACT at weeks 1 and 8 of the ASW-led phase and week 1–3 of the peer-led phase.

**Table 1.** PRIZE process evaluation results

Pre-condition	Indicator (data source)	Result	Illustrative quote
**P1: Service users and caregivers are identified and have initial interest in attending group sessions**	Number of service users and caregivers eligible and consenting to participate	17/201 (8.5%) potential participants ineligible. 84/184 (45.7%) eligible participants declined to participate.	-
	Reasons for declining	25/84 (29.8%) Groups not perceived to be relevant to needs 19/84 (22.6%) Did not have time to participate 14/84 (16.7%) Did not wish to be interviewed 11/84 (13.1%) Other reason 8/84 (9.5%) Did not wish to participate in group format 6/84 (7.1%) Venue was inaccessible 1/84 (0.5%) Missing data	
	IDIs with individuals declining to participate at recruitment	Lack of understanding on the purpose and nature of groups, and venue accessibility issues.	*‘I thought they’ll take me to an old age home … that’s why I didn’t agree to participate’* Male service user IDI
**P2: ASWs attend training**	Number training sessions attended	All ASWs attended 3 days initial training and majority of 29 weekly one-hour top-up training sessions.	-
**P3: Social worker supervises ASWs**	Number of weekly supervisions & monthly observations conducted.	Social worker conducted supervision fortnightly instead of weekly; all ASWs had 100% attendance. Social worker conducted fewer observations than planned (2/3 for one group, 1/3 for three groups, 0/3 for two groups).	-
	Perception of adequacy of supervision (IDIs with ASWs and social worker)	Supervision was perceived to be adequate by ASWs and SW. Key needs were met for problem solving on emerging issues and improving communication between co-facilitators. GroupACT contributed to enabling meaningful feedback.	*‘We knew that come Friday [supervision] we are going to be sorted. We use that feedback in the following sessions. That is how we improved every day’.* ASW IDI
**P4: ASWs have skills to successfully facilitate groups**	GroupACT scores from observations	Week 1 mean GroupACT score 2.9 (SD 2.3), indicating most basic skills observed. Lowest item scores: confidentiality, barriers to attendance and problem solving. Highest item scores: group participation, fostering empathy. Week 8 mean GroupACT score 4.0 (SD 2.3), indicating all basic skills and some advanced skills observed. Highest obtainable score for all items.	-
	(Self-) perception of facilitation skills and competence (IDIs with service user, caregiver & ASWs)	Peers viewed ASWs as having the necessary skills for facilitation. ASWs highlighted training and use of the facilitation guide built confidence and competence.	*‘The training was very informative and the role playing was one thing that prepared us more … training was so effective in the sense that it gave us confidence’.* ASW IDI
**P5: ASWs remind peers to attend**	% participant-sessions with attempted reminder	ASW-led phase: 92% participant-sessions (100% amongst participants attending ≥1 session) Peer-led phase: 78% participant-sessions (100% amongst participants attending ≥1 session)	-
	% attempted reminders successfully conveyed	ASW-led phase: 71% successfully conveyed (71% service users vs 72% caregivers). Range 63–78% between groups. Peer led phase: 67% successfully conveyed (63% service users vs 73% caregivers). Range 56–80% between groups.	-
**P6: Peers have interest and willingness to be facilitators**	Two peer facilitators identified for each group	Median 3 peer facilitators per group (range 1–4). Median 2 service users (range 0–3) and 1 caregiver (range 0–2).	-
	Barriers and motivators to taking peer facilitator role (FGDs with peer facilitators)	Barriers to taking on the role were lack of confidence and the belief that peers did not have the same knowledge and skills as ASWs. Motivators were the desire to share experiences and believing they could fulfil the role based on ASWs role modelling.	‘*I wanted to share my story about my life experiences as someone with mental illness and to show the dignity of a person living with mental illness’* Male service user, peer facilitator FGD
**P7: ASWs support peer facilitators**	% peer-led sessions shadowed by ASW	Mean 75.4% peer-led sessions shadowed by ASW (range by group 33.3–100%), compared to planned 38.5%	-
	Perception of adequacy of support received (FGDs with peer facilitators)	Peer facilitators valued rode modelling, mentoring and ongoing support (presence at sessions, follow-up calls).	*‘She [social worker] comes back to the group and she’ll ask if I managed, that’s support … they didn’t just hand over the group to us, there were there for us as well’* Female caregiver, peer facilitator FGD
**P8: ASWs refer participants to services in line with recovery plan**	Number of referrals made to Indlela Mental Health	6/46 (13.0%) service user-caregiver units had a referral (4 SUs and 2 CGs). Reasons: relapse (*n* = 1), issues with disability grant (*n* = 3), family relationship problems (*n* = 2)	-
	% of referrals resulting in service contact	100% referrals resulted in service contact	-
	Perception of whether referrals are in line with recovery plan (IDIs with service users)	ASWs referrals were beneficial in relation to management of side effects and applications for disability grants.	*‘The social workers referred me to Indlela Mental Health … and that letter made it possible for me to successfully apply for the disability grant’.* Male service user IDI
**P9: Peers have sense of group belonging and ownership**	Perception of belonging and ownership (IDIs with service users, caregivers and ASWs)	Peers described positive group dynamics and being comfortable participating and sharing. Groups developed as a trusting environment where peers felt they belonged and gained support.	*‘In the group we treat each other as family, we share about everything even the [auxiliary] social worker is part of our family’* Female service user IDI
**P10: Peers attend sessions regularly**	Number of sessions held (planned 9 ASW sessions and 13 peer led sessions)	ASW-led phase: All groups held 9 sessions Peer-led phase: median 10 sessions (range 8–13) held in 4-month project window (median 13.5 including sessions outside project window)	-
	% attendance at held sessions amongst all participants allocated to intervention arm	ASW-led phase: participants attended mean 59% sessions (service users 67% vs caregiver 46%). Range between groups 48–74%. Peer-led phase: participants attended mean 41% sessions (service users 47% vs caregiver 29%). Range between groups 17–59%.	-
	% attendance at held sessions amongst participants who attended ≥1 session/s	ASW-led phase: participants attended mean 65% sessions (service users 73% vs caregiver 54%). Peer led-phase: participants attended mean 44% sessions (service users 50% vs caregiver 34%).	
	IDIs with service users and caregivers	Reasons for non-attendance included distance from venue, engagement in other tasks (e.g. job seeking, collecting grant or medication), unavailability of refreshments and bad weather	*‘The only thing that I didn’t like about the group is that I had to walk a long distance … it takes me approximately thirty minutes’* Male service user IDI
**P11: Peers share personal experiences and coping strategies**	Perception of degree of sharing experiences/ strategies (IDIs with service users, caregivers and ASWs)	Group members described positive group dynamics and being comfortable participating and sharing with the group. Group problem solving enabled sharing of experiences and coping strategies.	*‘By sharing my stories and others shared theirs, I got support and became motivated. We got a chance to discuss and solve different problems … that enabled us to come up with realistic and doable solutions’* Male service user IDI
**P12: Peers develop personal recovery plan**	Perception of how engaged participants are in recovery planning (IDIs with service users, caregivers and ASWs)	Recovery planning motivated most group members. A minority felt stress around not being able to achieve goals and were less comfortable sharing.	*‘I share with others, but I don’t really like sharing about my goals before … I prefer sharing with others once I have managed to achieve my goal’.* Female service user IDI
**P13: Peers shape group focus to their priorities**	Number of external speakers	2 groups had 1 external speaker (local businessman) for 1 session each. 4 groups had 0 external speakers.	-
	Perception of degree of shaping to peer priorities (IDIs with service users, caregivers and ASWs)	Peers directed their group’s process by generating topics for group problem solving and contributing to these discussions. ASWs developed a respect for strengths of group members and their direction of the groups.	*‘Being a facilitator, you need to be open minded, let them [peers] teach you. You learn as you go along with them … it’s just to be yourself. and let them be themselves’.* ASW IDI
**P14: Peers solve problems to work towards recovery**	Perception of usefulness of ideas and information for recovery (IDIs with service users, caregivers)	Group problem solving had tangible benefits for recovery: increased knowledge, reduced loneliness, improved ability to cope with medication side effects, reduced alcohol consumption, and improvements in family relationships.	‘*I’ve noticed a great change in myself as I was not a vocal person, I was shy I couldn’t talk in front of many people … being part of the group enabled me to open up … It makes me feel good, because previously when I say something at home, they wouldn’t pay attention to what I’m saying. Now they consider what I say in the house, I am able to express myself’.* Female service user IDI
**P15: Caregivers develop strategies to support their relative**	(Self-) perception of caregiver strategies and skills (IDIs with service users, caregivers)	Caregivers comforted each other and shared experiences. This increased understanding and empathy for service users and improved caregivers’ ability to bring patience and support to their role.	*‘I have learned that I need to stand with her and walk with her through the journey and give her support’* Female caregiver IDI
**P16: All peers contribute to running of group**	Perception of peer contribution (IDIs with service users, caregivers and ASWs)	Peers felt they had sufficient opportunity to participate in sessions. Assisting with practical aspects of the group (e.g. leading prayers, assisting with refreshments) helped members see themselves as valued.	‘*I participated as I learnt from the stories. I got along with everyone. I shared about my illness … I used to sweep the venue … It made me feel better and motivated’.* Female service user IDI
**P17: Peer facilitators attend training**	% peer facilitators attending training	8/16 (5 service users, 3 caregivers) peer facilitators attended 1 of 4 planned half day training sessions. 16/16 received 1:1 training sessions immediately before/after groups.	-
**P18: Peer facilitators have skills to successfully facilitate groups**	GroupACT scores from observations	Week 1–3 mean GroupACT score 2.1 (SD 0.3), indicating some but not all basic skills observed. Lowest item scores: time management and confidentiality. Highest item scores: establishing ground rules, fostering empathy.	-
	(Self-) perception of facilitation skills and competence (IDIs with service users, caregivers and ASWs, FGDs with peer facilitators)	Some peer facilitators felt they had been upskilled to fulfil their role, but others desired further training and experience. For some peer facilitators their role had a positive influence on recovery promoting self-development and confidence.	*‘It was not as difficult as I thought it would be before … I participated fully as I’m the lead of the group so I had to set an example for other group members. I made sure that I cover everything in the right manner so that they can have confidence in me, so I prepared myself before each session’* Female *c*aregiver peer facilitator IDI
**P19: Recovery groups meet the economic needs of peers**	Perception of extent to which groups met economic needs (IDIs with service users, caregivers and ASWs, FGDs with peer facilitators)	Service users and caregivers expressed some unmet needs: desire for paid employment, skills building (handwork), physical activities and assistance with urgent problems (e.g. safe housing).	*‘Maybe invite someone to teach us about gardening, maybe starting small gardens in our homes … But I would like for PRIZE to create job opportunities’* Male service user IDI
**P20: Peers accept peer facilitators**	Perception of extent to which peer facilitators were acceptable to participants (IDIs with service users, caregivers and ASWs, FGDs with peer facilitators)	Peers felt a gap in motivation and direction of the group when the ASW was not present and they and ASWs linked this to the drop in participation. Some peers noted a lack of respect for peer facilitators.	*‘[the group members] don’t take the peer facilitators seriously … there is some sort of disrespect. Because at least when the social worker is present, they show respect, they don’t do as they please … It makes me feel bad emotionally not being respectful in the group’* Male Service user IDI

#### Outcome evaluation

Quantitative data for all outcomes were collected at baseline, 2 months and 5 months post-randomisation at the participant’s clinic or home. Service user outcomes were: functioning (self- and proxy-rated 12-item WHO Disability Assessment Schedule [WHODAS][Ustün *et al.*, 2010]), personal recovery (Recovery Assessment Scale-Domains and Stages [RAS-DS][Hancock *et al.*, 2014]), unmet needs (Camberwell Assessment of Need Short Assessment Schedule [Slade and Thornicroft, 2020]), internalized stigma (Internalized Stigma of Mental Illness Scale [ISMI][Ritsher *et al.*, 2003]), perception of respect and value (two questions based on formative work), alcohol use (Alcohol Use Disorders Identification Test-Consumption [AUDIT-C])(Morojele *et al.*, 2017), health service use (bespoke questions), relapse (hospitalisation or police contact due to mental health in last 2 months), and medication adherence (5-point ordinal scale). The caregiver outcome was caregiver burden (caregiving consequences of the Involvement Evaluation Questionnaire [IEQ][Van Wijngaarden *et al.*, 2000]). Support for recovery (Brief INSPIRE (Williams *et al.*, 2015)) was assessed in intervention arm participants only, in relation to their ASW facilitator (2 months), peer facilitator (5 months) and psychiatric nurse (baseline, 2 and 5 months) (Supplementary file 3). All instruments were translated into isiXhosa and back-translated to English to check for semantic equivalence. Cognitive interviewing was carried out for the WHODAS, CANSAS and RAS-DS. Study data were collected and managed on Android tablets using REDCap electronic data capture tools (Harris *et al.*, 2019). Attrition from the study was minimised through phone/text reminders. Serious adverse events (SAE), including death and hospitalisation, were detected through participants informing (i) the assessor at data collection, (ii) the ASW at recovery groups or (iii) the trial coordinator by telephone. Assessors and ASWs informed the trial coordinator, who confirmed SAE details by contacting the service user and/or caregiver.

#### Assessment of trial procedures

The proportions consenting to participate and lost to study follow up were recorded. To assess for contamination at the 5-month endpoint, all control arm participants were asked about knowledge of, and attendance to, recovery groups.

### Data analysis

Thematic analysis of qualitative data was conducted using NVivo 12 to manage the data (QSR, 2020). A deductive approach was used to map data to the theory of change preconditions, whilst an inductive process was used to identify additional themes (Proudfoot, 2023). A descriptive analysis of quantitative process indicators was undertaken. The outcome analysis was completed using Stata 15.0 (Statacorp, 2015). The relapse variable was derived from endpoint interview self-report data and SAE data relating to hospitalization. This allowed us to include relapse data for all participants, including those who did not complete endpoint interviews. A sensitivity analysis was conducted to exclude individuals who had died or withdrawn, to avoid misclassification of relapse status. To estimate the potential effect of recovery groups at 2 and 5 months, quantitative outcomes were compared between treatment arms, adjusting for baseline scores and clinic, using linear mixed models for continuous variables and generalized linear mixed models for binary variables based on an intention-to-treat analysis. To assess differences in support for recovery between service providers, the paired t-test was used to compare Brief INSPIRE scores amongst intervention arm participants between facilitator types at each relevant time point. We analysed the data using an available case analysis, that is all individuals providing data for any outcome at any timepoint were included.

## Results

Between 16 May 2022 and 7 September 2022, a total of 201 individuals were identified at clinics and underwent initial eligibility assessment, of whom 68 were excluded at this stage (50 declined to participate)(See Fig 1). Of the 133 individuals who underwent full eligibility assessment, 41 individuals were excluded (34 declined to participate and 7 lacked capacity). The most common reasons for declining to participate at recruitment were perceiving groups to be irrelevant to needs (25 of 84 decliners) and not having time (19 of 84 decliners) (see Table 1). Of the 92 service users randomised, 46 service users (and 19 linked caregivers) were randomised to TAU and 46 service users (and 27 linked caregivers) were randomised to recovery groups plus TAU. Of these, 81 service users (88.0%) completed the 5-month follow-up assessment. Thirteen percent of service users in the control arm were aware of the recovery groups but none had participated. Table 2 presents baseline demographic and clinical characteristics by treatment arm. The majority of caregivers were parents and siblings.

**Table 2.** Baseline characteristics by treatment arm

	Treatment as usual	Recovery group and treatment as usual	Total
**Service users**	***N* = 46**	***N* = 46**	***N* = 92**
**Sex (*n* [%])**			
Male	35 (76%)	33 (72%)	68 (74%)
Female	11 (24%)	13 (28%)	24 (26%)
**Age (years) (mean [SD])**	46.6 (12.1)	44.7 (11.1)	45.6 (11.6)
**Marital status (*n* [%])**			
Married	1 (2%)	3 (7%)	4 (4%)
Widow/widower	1 (2%)	3 (7%)	4 (4%)
Divorced or separated	0 (0%)	3 (7%)	3 (3%)
Never married (single)	44 (96%)	37 (80%)	81 (88%)
**Employment status (*n* [%])**			
Unemployed and looking for work	15 (33%)	20 (43%)	35 (38%)
Unemployed and not looking for work	26 (57%)	24 (52%)	50 (54%)
Employed part-time	4 (9%)	1 (2%)	5 (5%)
Pensioner	1 (2%)	1 (2%)	2 (2%)
**Education status (*n* [%])**			
Primary education	13 (28%)	18 (39%)	31 (34%)
Secondary education	28 (61%)	24 (52%)	52 (57%)
Diploma/degree	5 (11%)	4 (9%)	9 (10%)
**Problems with learning (*n* [%])**			
No	36 (78%)	36 (78%)	72 (78%)
Yes	10 (22%)	10 (22%)	20 (22%)
**Living situation (*n* [%])**			
I have a place to live where I can stay as long as I want	44 (96%)	46 (100%)	90 (98%)
I currently have a place to live, but may not be able to stay there in the future	2 (4%)	0 (0%)	2 (2%)
**Main source of income (*n* [%])**			
Odd jobs	2 (4%)	5 (11%)	7 (8%)
Government disability grant	39 (85%)	35 (76%)	74 (80%)
No income	5 (11%)	4 (9%)	9 (10%)
Other	0 (0%)	2 (4%)	2 (2%)
**Monthly income (*n* [%])**			
Less than R600 (32 USD)	8 (17%)	8 (17%)	16 (17%)
R600–1000 (32–53 USD)	0 (0%)	1 (2%)	1 (1%)
R1001–2000 (54–107 USD)	33 (72%)	34 (74%)	67 (73%)
R2001–4000 (108–213 USD)	4 (9%)	3 (7%)	7 (8%)
Don’t know	1 (2%)	0 (0%)	1 (1%)
Self-reported total WHODAS (mean [SD]*)	8.2 (8.2)	7.9 (11.3)	8.0 (9.8)
**Relapse in last 2 months (*n* [%])**			
No	44 (96%)	43 (94%)	88 (96%)
Yes	2 (4%)	3 (7%)	5 (5%)
**Internalized stigma total score (mean [SD])**	2.3 (0.6)	2.5 (0.7)	2.4 (0.7)
**Recovery (RAS-DS) total score (mean [SD])**	89.0 (16.9)	89.9 (17.6)	89.4 (17.2)
**Number of unmet needs (CANSAS) (mean [SD])**	1.6 (1.1)	1.5 (1.7)	1.5 (1.4)
**Contact with mental health nurse last 2 months (*n* [%])**			
No	0 (0%)	2 (4%)	2 (2%)
Yes	46 (100%)	44 (96%)	90 (98%)
**Antipsychotic medication adherence (*n* [%])**			
All the time	46 (100%)	45 (98%)	91 (99%)
Most of the time (>3 of the last 4 weeks)	0 (0%)	1 (2%)	1 (1%)
Sometimes, occasionally, or not at all	0 (0%)	0 (0%)	0 (0%)
**AUDIT-C total ≥3 (female) or ≥4 (male) (*n* [%])**			
No	37 (80%)	38 (83%)	75 (82%)
Yes	9 (20%)	8 (17%)	17 (18%)
**Caregivers**	***N* = 19**	***N* = 28**	***N* = 47**
**Relationship of caregiver to service user**			
Parent	6 (32%)	8 (29%)	14 (30%)
Sibling	4 (21%)	10 (36%)	14 (30%)
Other family member	2 (10%)	5 (18%)	7 (15%)
Child	3 (16%)	1 (4%)	4 (8%)
Partner	1 (5%)	3 (11%)	4 (8%)
Friend	3 (16%)	1 (4%)	4 (8%)
**Caregiver burden mean IEQ score (SD)**	17.9 (2.7)	16.2 (2.2)	16.9 (1.7)

### Process evaluation

The majority of pre-conditions were met (Table 1), indicating broad acceptability and feasibility of the intervention. Service user and caregiver peers described positive group dynamics and being comfortable sharing with the group (P9). Groups were seen as a chance for ‘lightness’, feeling hopeful and motivated. Refreshments reportedly enhanced the appeal of groups. Mixed service user/caregiver groups were acceptable to all participants, with the benefit of increasing service users’ sense of inclusion, opportunities for understanding each other and joint problem solving (P11, P15). Recovery planning and goal setting left most group members motivated. A minority felt stress caused by not being able to achieve goals, often due to financial barriers (P12). Assisting with practical aspects of the group (e.g. leading prayers) helped members see themselves as valued (P13). Sharing experiences and group problem solving reportedly led to some tangible benefits, including improved knowledge, ability to cope with medication side-effects, self-esteem, ability to manage debts and strengthened communication and relationships within families; and reduced loneliness, alcohol consumption and stress (P14). For some peer facilitators their role had a positive influence on recovery, giving a sense of self-development, and confidence in being able to express feelings. ASWs’ commitment to groups, facilitation skills and onward referrals (e.g. for assistance on applying for disability grants) were highly valued (P4, P8). The GroupACT was valued for providing the opportunity for meaningful feedback (P3).

Five pre-conditions were not fully met. First, social worker supervision was less frequent than planned (P3), though this did not appear to impact ASW competence, with all ASWs demonstrating advanced skills by the endpoint (Table 1 and Supplementary File 4). Second, although participant reminders were valued and largely attempted as planned, only two thirds were successfully conveyed (i.e. ASW spoke with participant) (P5). Third, not all group members attended sessions regularly: amongst all participants randomised to recovery groups, a mean of 59% and 41% sessions were attended in the ASW-led and peer-led phases, respectively (65% and 44% amongst participants who attended ≥1 session/s) (P10). Attendance was lower amongst caregivers in both phases. Attendance varied considerably between groups (17–59% in the peer-led phase). A key facilitator of success was the presence of motivated individuals, who exerted a powerful ripple out effect influencing other group members. Practical reasons for non-participation included distance from the venue (and lack of transport money), caregivers looking for employment or having other caregiving responsibilities, service users collecting disability grants or treatment and bad weather. Fourth, the reconfiguration to avoid large group training sessions meant peer facilitators received less training than planned (P17). Several peer facilitators desired more training and support. Finally, peer facilitators did not always have the confidence and skills to facilitate the groups alone (P18). GroupACT scores at baseline of peer-led groups indicated peer facilitators demonstrated some but not all basic skills (Supplementary File 4). Due to requests from group members and peer facilitators, ASWs shadowed approximately twice as many peer-led sessions as planned (P7).

We identified two additional pre-conditions which are needed for the intervention to function (specifically to promote participation), and which were not fully met. First, groups should meet the economic needs of participants (P19). Peers highlighted some critical needs that were not met by groups, including the reduction of financial instability (including support to access paid employment), skills development e.g. ‘handwork’, and assistance with urgent problems (e.g. accessing safe housing). Second, peer facilitators should be acceptable to group members (P20). Some peer facilitators felt group members were disrespectful and undermined them. Group members commonly felt a gap in motivation and direction of the group when the ASW was not present and they, ASWs and peer facilitators linked this to the attendance drop. This decrease in collective focus was compounded by the unavailability of refreshments in the peer-led phase.

### Outcome evaluation

Recovery groups appeared to positively impact on relapse. Relapse occurred in 1 (2.2%) of 46 participants in the recovery group arm compared to 8 (17%) of 46 participants in the control arm (risk difference  −0.15 [95% CI: −0.26; −0.05]) (Table 3). There was no change in the effect when two individuals who had died and withdrawn were excluded. Recovery groups also appeared to impact on the proxy-reported number of days in the last month service users were totally unable to work (mean 1.4 days recovery group arm vs 7.7 days control arm; adjusted mean difference −6.3 [95%CI: −12.2; −0.3]). No impacts were detected at 5 months on other functioning markers, personal recovery, unmet needs, internalized stigma, perception of respect and value, alcohol use, health service use, medication adherence or caregiver burden (Table 3). No impacts were detected on any outcome at 2 months (Supplementary File 5). Service users in the intervention arm reported significantly greater support for recovery from ASW facilitators compared to mental health nurses at 2 months. No difference in recovery support was detected between ASWs and peer facilitators, nor between peer facilitators and mental health nurses at 5 months (Table 4). Those who completed 5-month follow up had better medication adherence than those who were lost to follow up (Supplementary File 6). Participating in recovery groups appeared to exert a stronger effect on relapse amongst service users without a caregiver compared to those with a caregiver (Supplementary File 7). There was one death and one hospitalisation in the recovery group arm and eight hospitalisations in the control arm.

**Table 3.** PRIZE 5-month outcome evaluation results

	Treatment as usual (*n* = 39)	Recovery groups and treatment as usual (*n* = 42)	Mean difference or risk difference (95% CI)
**Disability**			
**Self-reported total WHODAS (mean [SD])**	5.8 (4.4)	7.3 (9.8)	1.55 (−2.04; 5.14)^a^
**Self-reported days totally unable to work (mean [SD])**	0.1 (0.3)	0.4 (2.3)	0.31 (−0.59; 1.20)^a^
**Self-reported days reduced ability to work (mean [SD])**	0.3 (1.6)	0.4 (1.7)	0.09 (−0.84; 1.02)^a^
**Proxy-reported total WHODAS (mean [SD])**	10.3 (12.4)	9.5 (15.0)	−0.03 (−6.03; 5.97)^a^
**Proxy-reported days totally unable to work (mean [SD])**	7.7 (12.5)	1.4 (4.4)	−6.25 (−12.18; −0.31)^a^
**Proxy-reported days reduced ability to work (mean [SD])**	2.4 (7.5)	2.4 (6.2)	0.58 (−1.48; 2.64)^a^
**Relapse**			
**Hospitalisation or police contact in last 2 months (interview and SAE data) (*n* [%]) (*n* = 92)**	8 (17.4%)	1 (2.2%)	−0.15 (−0.26; −0.05)^b^
**Health service use**			
**No contact with mental health nurse last 2 months (*n* [%])**	0	1 (2%)	−0.024 (−0.070; 0.022)^b^
**Stigma**			
**Internalized stigma (ISMI) mean score (SD)**	1.8 (0.6)	1.9 (0.6)	−0.03 (−0.3; 0.24)^a^
**Does not feel valued and respected by family (*n* [%])**	1 (3%)	3 (7%)	0.13 (−0.52; 0.79)^a^
**Does not feel valued and respected by community (*n* [%])**	4 (10%)	4 (10%)	0.02 (−0.03; 0.07)^a^
**Recovery**			
**RAS-DS total score (mean [SD])**	84.9 (10.7)	85.6 (11.3)	0.52 (−3.13; 4.16)^a^
**Unmet needs**			
**Number of unmet needs (CANSAS) (mean [SD])**	1.5 (1.4)	1.5 (1.3)	−0.004 (−0.942; 0.934)^a^
**Medication adherence**			
**Non-adherent to antipsychotic medication (*n* [%])**	0 (0%)	1 (2%)	−0.024 (−0.070; 0.022)^b^
**Hazardous drinking**			
**AUDIT-C total ≥3 (female) or ≥4 (male) (*n* [%])**	7 (18%)	8 (19%)	−0.01 (−0.12; 0.11)^a^
**Caregiver burden**	***n* = 17**	***n* = 23**	
**Total IEQ score (mean [SD])**	14.6 (17.5)	11.0 (7.3)	−2.73 (−11.0; 5.54)^a^

a Adjusted for baseline score of outcome variable and clinic

b Unadjusted analysis due to low numbers

**Table 4.** Comparison of Brief INSPIRE scores between time points and facilitator types

	Mean Brief INSPIRE^a^ (SE)				
	2 months		5 months		
Comparison (n)	Mental health nurse	ASW facilitator	Mental health nurse	Peer facilitator	Paired T test *p* value
**Mental health nurse 2 months & ASW facilitator 2 months (*n* = 35)**	60.3 (4.4)	74.8 (1.7)	-	-	*p* < 0.001
**Mental health nurse 5 months & peer facilitator 5 months (*n* = 29)**	-	-	74.3 (4.4)	68.6 (5.5)	0.27
**ASW facilitator 2 months & peer facilitator 5 months (*n* = 27)**	-	74.7 (2.1)	-	70.6 (5.3)	0.43

a Scale 0–100; higher scores indicate greater support for recovery

## Discussion

This mixed-methods study assessed the acceptability, feasibility and potential effectiveness of recovery groups for people with psychosis including peers as facilitators, through a randomised feasibility trial. Overall, we demonstrated the feasibility of implementing this complex mental health intervention in partnership with a grassroots NGO in a low resource South African setting. The wide variation in attendance between groups suggests some worked well whilst others did not. For attenders, groups were an enjoyable and hopeful space and a chance for positive social interactions. Feasibility and acceptability were most clearly demonstrated in the ASW-led phase, and participants reported superior recovery support from ASWs compared to mental health nurses. Whilst peer facilitators themselves experienced the role as an opportunity to flourish in terms of self-confidence, some group members found the peer-led phase less satisfactory. However, despite not being powered to detect intervention effects, there were promising indications that groups could reduce relapse rates. This suggests that regular supportive contact with peers, and specific strategies that individuals developed to promote their wellbeing, had meaningful effects which extended beyond the groups. There was some indication that those without existing social support (in the form of a caregiver able to attend) may benefit the most from the groups. Good recruitment and retention rates point to the feasibility of conducting a full trial. A strength of this study was the use of theory of change to structure the evaluation. Exploring whether preconditions were met gives a clear picture of potential reasons why recovery groups did not have a greater impact on outcomes such as personal recovery and allows us to make specific recommendations to increase the likelihood of impact. Important limitations were the lack of endline GroupACT data for peer facilitators, and the absence of a measure of personal recovery designed for the South African setting. There were very low numbers reporting health service non-engagement and medication non-adherence. Future evaluations should consider the utility of such outcomes and/or alternative measures.

In common with peer support evaluations in Chile, Uganda and Tanzania, some participants were reluctant to accept support from peers as they were not deemed to be hierarchically superior. However, in PRIZE the perceived inferiority was primarily related to the facilitators’ lack of professional qualifications (Le *et al.*, 2022) rather than their mental health (Ramesh *et al.*, 2023). Our formative findings supported the acceptability of service users and caregivers assuming the role of group facilitator. We suggest that once support from ASWs (trained professionals) had been experienced by participants in the trial (in a context where this is not usually available), the shift to peer facilitation was perceived as a gap. The relatively light touch training delivered to peer facilitators was designed to be scalable in low resource settings, as well as responsive to peer facilitators who found large group training inaccessible. The INSPIRE data suggests peer and ASW facilitators offered similar levels of support for recovery. However, our qualitative results suggest the final training package was inadequate for group members and peer facilitators to have confidence in their skills. To address these concerns, we recommend that future similar interventions should avoid a two-phase model. Instead, potential peer facilitators should be identified from the outset and begin a co-facilitation role early on. Crucially, structured support from ASWs should continue for the duration of the intervention, rather than tailing off. To maintain harmony amongst peers, and to maximise intervention scalability, peer facilitators were not paid for their role. Compensating lived experience expertise might more clearly signal peers’ status as trained facilitators, as well as addressing the human rights imperative (Sartor, 2023).

Lack of opportunities for increasing financial security were important acceptability issues across phases, despite some participants accessing government disability grants, and poverty was itself a barrier to attending groups. Economic interventions such as cash transfers can play a role in alleviating depression (Wollburg *et al.*, 2023), and a Kenyan cohort study demonstrated benefits of savings groups on functioning amongst people with psychosis (Lund *et al.*, 2013). However, randomized evaluations of economic interventions for people with psychosis are scarce in LMIC (Joyce Protas *et al.*, 2022). Future recovery group models could incorporate practical productive activities, and approaches to improve financial stability, such as savings groups.

A third of attempted reminders were not successfully conveyed, typically because of lack of phone ownership or airtime in participants, and conceivably contributing to low attendance. Future implementation could consider home visit reminders, which could also encourage a sense of inclusion. Potential benefits of this approach should be balanced with workforce considerations. Communal eating can be an important part of personal recovery (Vogel *et al.*, 2019). Provision of refreshments was an important draw for PRIZE participants. To maximize intervention scalability participants were encouraged to self-organise refreshments in the peer-led phase. However, due to high poverty levels this was not successful, and the absence of refreshments reportedly contributed to attendance decreasing. Future models should prioritize ongoing refreshment provision working with local NGO providers to enable sustainability.

In conclusion we have demonstrated encouraging findings relating to the acceptability and feasibility of supported PRIZE. Our findings are generalisable to other LMICs. A larger definitive trial, incorporating our recommendations to enhance acceptability and feasibility, is needed to demonstrate intervention effectiveness.

## Supporting information

Asher et al. supplementary material 1 Asher et al. supplementary material

Asher et al. supplementary material 2 Asher et al. supplementary material

Asher et al. supplementary material 3 Asher et al. supplementary material

Asher et al. supplementary material 4 Asher et al. supplementary material

Asher et al. supplementary material 5 Asher et al. supplementary material

Asher et al. supplementary material 6 Asher et al. supplementary material

Asher et al. supplementary material 7 Asher et al. supplementary material

## Data Availability

The data and PRIZE intervention materials are available from the corresponding author on reasonable request.

## Supplementary material

The supplementary material for this article can be found at https://doi.org/10.1017/S2045796024000556.
