# Peer-led recovery groups for people with psychosis in South Africa (PRIZE): results of a randomized controlled feasibility trial – CORRIGENDUM

**DOI:** 10.1017/S2045796025000253

**Published:** 2025-05-14

**Authors:** Laura Asher, Bongwekazi Rapiya, Julie Repper, Tarylee Reddy, Bronwyn Myers, Gill Faris, Inge Petersen, Charlotte Hanlon, Carrie Brooke-Sumner

The authors regret the inclusion of errors in the Methods and Results Section (Outcome Evaluation) and Table 3. The correct information is shown here.

1. The authors would like to specify that the relapse variable was derived from 2-month and 5-month endpoint interview self-report data and serious adverse event (SAE) data relating to hospitalization.
2. The paper wrongly stated that ‘Relapse occurred in 1(2.2%) of 46 participants in the recovery group arm compared to 8 (17%) of 46 participants in the control arm (risk difference -0.15 (95% CI -0.26;-0.05))’. The authors would like to correct this to ‘Relapse occurred in 1 (2.2%) of 46 participants in the recovery group arm compared to 9 (19.6%) of 46 participants in the control arm (risk difference, −0.17 [95% CI: −0.30; −0.04])’.
3. The abstract containing the correction of the relapse outcome data is as follows

**Abstract**

**Aims.** The aims of this feasibility trial were to assess the acceptability and feasibility of peer-led recovery groups for people with psychosis in a low-resource South African setting, to assess the feasibility of trial methods and to determine key parameters in preparation for a definitive trial.

**Methods.** The design was an individually randomized feasibility trial comparing recovery groups in addition to treatment as usual (TAU) with TAU alone. Ninety-two isiXhosa-speaking people with psychosis, and forty-seven linked caregivers, were recruited from primary care clinics and randomly allocated to trial arms in a 1:1 allocation ratio. TAU comprised anti-psychotic medication delivered in primary care. The intervention arm comprised six recovery groups including service users and caregivers. Two-hour recovery group sessions were delivered weekly in a 2-month auxiliary social worker (ASW)-led phase, then a 3-month peer-led phase. To explore acceptability and feasibility, a mixed methods process evaluation included 25 in-depth interviews and 2 focus group discussions at 5 months with service users, caregivers and implementers, and quantitative data collection including attendance and facilitator competence. To explore potential effectiveness, quantitative outcome data (functioning, relapse, unmet needs, personal recovery, stigma, health service use, medication adherence and caregiver burden) were collected at baseline, 2 months and 5 months post-randomization. Trial registration: PACTR202202482587686.

**Results.** Qualitative interviews revealed that recovery groups were broadly acceptable with most participants finding groups to be an enjoyable opportunity for social interaction and joint problem-solving. Peer facilitation was a positive experience; however, a minority of participants did not value expertise by lived experience to the same degree as expertise of professional facilitators. Attendance was moderate in the ASW-led phase (participants attended 59% sessions on average) and decreased in the peer-led phase (41% on average). Participants desired a greater focus on productive activities and financial security. Recovery groups appeared to positively impact on relapse. Relapse occurred in 1 (2.2%) of 46 participants in the recovery group arm compared to 9 (19.6%) of 46 participants in the control arm (risk difference, −0.17 [95% CI: −0.30; −0.04]). Recovery groups also impacted on the number of days in the last month totally unable to work (mean 1.4 days recovery groups vs 7.7 days control; adjusted mean difference, −6.3 [95% CI: −12.2; −0.3]). There were no effects on other outcomes.

**Conclusions.** Peer-led recovery groups for people with psychosis in South Africa are potentially acceptable, feasible and effective. A larger trial, incorporating amendments such as increased support for peer facilitators, is needed to demonstrate intervention effectiveness definitively.

4. As such Table 3 should be reported as follows

**Table 3.** PRIZE 5-month outcome evaluation results

	Treatment as usual (*n* = 39)	Recovery groups and treatment as usual (*n* = 42)	Mean difference or risk difference (95% CI)
**Disability**			
**Self-reported total WHODAS (mean [SD])**	5.8 (4.4)	7.3 (9.8)	1.55 (−2.04; 5.14)^a^
**Self-reported days totally unable to work (mean [SD])**	0.1 (0.3)	0.4 (2.3)	0.31 (−0.59; 1.20)^a^
**Self-reported days reduced ability to work (mean [SD])**	0.3 (1.6)	0.4 (1.7)	0.09 (−0.84; 1.02)^a^
**Proxy-reported total WHODAS (mean [SD])**	10.3 (12.4)	9.5 (15.0)	−0.03 (−6.03; 5.97)^a^
**Proxy-reported days totally unable to work (mean [SD])**	7.7 (12.5)	1.4 (4.4)	−6.25 (−12.18; −0.31)^a^
**Proxy-reported days reduced ability to work (mean [SD])**	2.4 (7.5)	2.4 (6.2)	0.58 (−1.48; 2.64)^a^
**Relapse**			
**Hospitalization or police contact in last 5 months (interview and SAE data) (*n* [%]) (*n* = 92)**	9 (19.6%)	1 (2.2%)	−0.17 (−0.30; −0.04)^b^
**Health service use**			
**No contact with mental health nurse last 2 months (*n* [%])**	0	1 (2%)	−0.024 (−0.070; 0.022)^b^
**Stigma**			
**Internalized stigma (ISMI) mean score (SD)**	1.8 (0.6)	1.9 (0.6)	−0.03 (−0.3; 0.24)^a^
**Does not feel valued and respected by family (*n* [%])**	1 (3%)	3 (7%)	0.13 (−0.52; 0.79)^a^
**Does not feel valued and respected by community (*n* [%])**	4 (10%)	4 (10%)	0.02 (−0.03; 0.07)^a^
**Recovery**			
**RAS-DS total score (mean [SD])**	84.9 (10.7)	85.6 (11.3)	0.52 (−3.13; 4.16)^a^
**Unmet needs**			
**Number of unmet needs (CANSAS) (mean [SD])**	1.5 (1.4)	1.5 (1.3)	−0.004 (−0.942; 0.934)^a^
**Medication adherence**			
**Non-adherent to antipsychotic medication (*n* [%])**	0 (0%)	1 (2%)	−0.024 (−0.070; 0.022)^b^
**Hazardous drinking**			
**AUDIT-C total ≥3 (female) or ≥4 (male) (*n* [%])**	7 (18%)	8 (19%)	−0.01 (−0.12; 0.11)^a^
**Caregiver burden**	***n* = 17**	***n* = 23**	
**Total IEQ score (mean [SD])**	14.6 (17.5)	11.0 (7.3)	−2.73 (−11.0; 5.54)^a^

a Adjusted for baseline score of outcome variable and clinic

b Unadjusted analysis due to low numbers

The authors apologize for the error.
