# Supplementary material for: Peer-led recovery groups for people with psychosis in South Africa (PRIZE): Results of a randomized controlled feasibility trial
Source: Epidemiol Psychiatr Sci. 2024 Oct 11;33:e47. doi: 10.1017/S2045796024000556 (PMC11561686; doi:10.1017/S2045796024000556)
Supplement: Asher et al. supplementary material 1 — Asher et al. supplementary material [file S2045796024000556sup001.docx]

**PRIZE Recovery Group Session Outlines**

**Session 1 Welcome and introduction to the PRIZE Recovery Group**

|  | Step | Focus | Time |
| --- | --- | --- | --- |
| Step 1 | Set up | - Setting up a welcoming environment - Observe all Covid protocols | 10 min |
| Step 2 | Welcome and introductions | - Getting to know one another - Explain the purpose of the Sessions | 15 min |
| Step 3 | Let’s be together exercise | - Discussing and agreeing on what behaviours will help everyone to work together | 10 min |
| Step 4 | Understanding mental health and recovery | - Anna and Mpumi’s story to help understand what mental health - Understanding what recovery is all about | 10 min |
| Step 5 | How the PRIZE recovery groups will work… | - Group members will know what will happen during the Sessions - Group members will know what topics are going to be discussed - Group members will be informed that this group will continue after the 9 Sessions are complete | 20 min |
| Step 6 | Relaxation exercise | - Teach a self-help skill | 5 min |
| Step 7 | Closing the session | - Wrap up session and prepare for next Session | 10 min |
| Step 8 | Informal socialising and clearing up | - Have fun and socialising together - Leave the building as you would want to find it! | 35 min |

**Session 2 Understanding mental health**

|  | Step | Focus | Time |
| --- | --- | --- | --- |
| Step 1 | Set up | - Setting up a welcoming environment - Observe all Covid protocols | 10 min |
| Step 2 | Parallel check in | - Two check-in groups – caregivers and service users, each with their own facilitator | 20 min |
| Step 3 | Understanding severe mental illness | - Understanding severe mental illness - Discuss causes and treatment and treatment side effects | 45 min |
| Step 4 | Relaxation exercise | - Learning a breathing technique | 5 min |
| Step 5 | Closing the session | - Wrap up session and prepare for next Session | 5 min |
| Step 6 | Informal socialising and clearing up | - Have fun and socialising together - Leave the building as you would want to find it! | 35 min |

**Session 3 Building self-esteem**

|  | Step | Focus | Time |
| --- | --- | --- | --- |
| Step 1 | **Set up** | - Setting up a welcoming environment - Observe all Covid protocols | 10 mins |
| Step 2 | Parallel check in and breathing exercise | - Two check in groups – caregivers and service users, each with their own facilitator - The Session more time is spent in this Step to provide an opportunity for caregivers to have an in-depth look at their own stressors - Use breathing exercise from last Session | 30 mins |
| Step 3 | Mental illness and self-esteem | - Share and discuss stories on stigma and self esteem - Systematically work through the activities provided to help group members to develop/strengthen their self-esteem | 55 mins |
| Step 4 | Closing the session | - Wrap up session and prepare for next Session | 5 mins |
| Step 5 | Informal socialising and clearing up | - Have fun and socialising together - Leave the building as you would want to find it! | 20 mins |

**Session 4 Recovery planning – Part 1 My personal recovery plan**

|  | Step | Focus | Time |
| --- | --- | --- | --- |
| Step 1 | Set up | - Setting up a welcoming environment - Observe all Covid protocols | 10 mins |
| Step 2 | Parallel check in | - Two check in groups – caregivers and service users, each with their own facilitator | 20 mins |
| Step 3 | Introduction to what recovery means | - Use Ncumisa’s story to help explain what recovery means | 15 mins |
| Step 4 | Sharing experiences and beginning a personal recovery plan | - Systematically work through the instructions to help group members to develop their own recovery plans | 30 mins |
| Step 5 | Relaxation exercise | - Tensing and relaxing muscles to help stay calm | 10 mins |
| Step 6 | Closing the session | - Wrap up session and prepare for next Session | 5 mins |
| Step 7 | Informal socialising and clearing up | - Have fun and socialising together - Leave the building as you would want to find it! | 30 mins |

**Session 5 Recovery planning – Part 2 When things aren’t going well**

|  | Step | Focus | Time |
| --- | --- | --- | --- |
| Step 1 | Set up | - Setting up a welcoming environment - Observe all Covid protocols | 10 mins |
| Step 2 | Parallel check in and breathing exercise | - Two check in groups – caregivers and service users, each with their own facilitator - The Session more time is spent in this Step to provide an opportunity for caregivers to have an in-depth look at their own stressors - Use breathing exercise from last session | 20 mins |
| Step 3 | Beginning a personal recovery plan | - My plans for managing ups and downs - My plans for managing a crisis | 35 mins |
| Step 4 | Support network | - Activity 3 | 10 mins |
| Step 5 | Relaxation exercise | - If there is time | 10 mins |
| Step 6 | Closing the session | - Wrap up session and prepare for next Session | 5 mins |
| Step 7 | Informal socialising and clearing up | - Have fun and socialise together - Leave the building as you would want to find it! | 30 mins |

**Session 6 Recovery planning – Part 3 Dreams and goals**

|  | Step | Focus | Time |
| --- | --- | --- | --- |
| Step 1 | Set up | - Setting up a welcoming environment - Observe all Covid protocols | 10 mins |
| Step 2 | Parallel check in | - Two check in groups – caregivers and service users, each with their own facilitator | 20 mins |
| Step 3 | Dreams and goals | - Identifying dreams and goals and possible barriers to achieving success | 30 mins |
| Step 4 | Setting goals and reaching our dreams | - Practical exercise to help with goal setting | 45 mins |
| Step 5 | Relaxation exercise | - Choosing a relaxation method that will help the group to focus and be calm | 10 mins |
| Step 6 | Closing the session | - Wrap up session and prepare for next Session | 5 mins |
| Step 7 | Informal socialising and clearing up | - Have fun and socialising together - Leave the building as you would want to find it! | 30 mins |

**Session 7 Thinking about money**

|  | Step | Focus | Time |
| --- | --- | --- | --- |
| Step 1 | Set up | - Setting up a welcoming environment - Observe all Covid protocols | 10 mins |
| Step 2 | Parallel check in | - Two check in groups – caregivers and service users, each with their own facilitator | 20 mins |
| Step 3 | Understanding the impact of money and debt | - Understand the impact of triggers and lifestyle on dealing with finances | 15 mins |
| Step 4 | Problem management to help manage money and prevent debt | - Understand simple principles about budgeting - Learn a problem management skill | 50 mins |
| Step 5 | Closing the session | - Wrap up session and prepare for next Session | 5 mins |
| Step 6 | Informal socialising and clearing up | - Have fun and socialising together - Leave the building as you would want to find it! | 20 mins |

**Session 8 Healthy relationships**

|  | Step | Focus | Time |
| --- | --- | --- | --- |
| Step 1 | Set up | - Setting up a welcoming environment - Observe all Covid protocols | 10 mins |
| Step 2 | Parallel check in | - Two check in groups – caregivers and service users, each with their own facilitator | 20 mins |
| Step 3 | Understanding healthy relationships | - Look at what makes up healthy and unhealthy relationships | 20 mins |
| Step 4 | Setting boundaries and communicating with confidence | - Learn about boundaries so that needs can be established and communication can be improved | 30 min |
| Step 5 | Relaxation exercise | - Tensing and relaxing muscles to help stay calm | 10 min |
| Step 6 | Closing the session | - Wrap up session and prepare for next Session | 10 min |
| Step 7 | Informal socialising and clearing up | - Have fun and socialising together - Leave the building as you would want to find it! | 20 min |

**Session 9 Celebrating our journey so far and next steps together**

|  | Step | Focus | Time |
| --- | --- | --- | --- |
| Step 1 | Set up | - Setting up a welcoming environment - Observe all Covid protocols | 10 min |
| Step 2 | Grateful for the lessons we learnt and grateful for each other! | - Share experiences and learnings of the past few weeks | 25 min |
| Step 3 | Certificate ceremony | - Celebrate each one for their contribution to the success of the group. | 25 min |
| Step 4 | Looking forward to next steps together | - prepare for the for next phase | 25 min |
| Step 5 | Informal socialising and clearing up | - Have fun and socialising together - Leave the building as you would want to find it! | 40 min |
