# Supplementary material for: Peer-led recovery groups for people with psychosis in South Africa (PRIZE): Results of a randomized controlled feasibility trial
Source: Epidemiol Psychiatr Sci. 2024 Oct 11;33:e47. doi: 10.1017/S2045796024000556 (PMC11561686; doi:10.1017/S2045796024000556)
Supplement: Asher et al. supplementary material 2 — Asher et al. supplementary material [file S2045796024000556sup002.pdf]

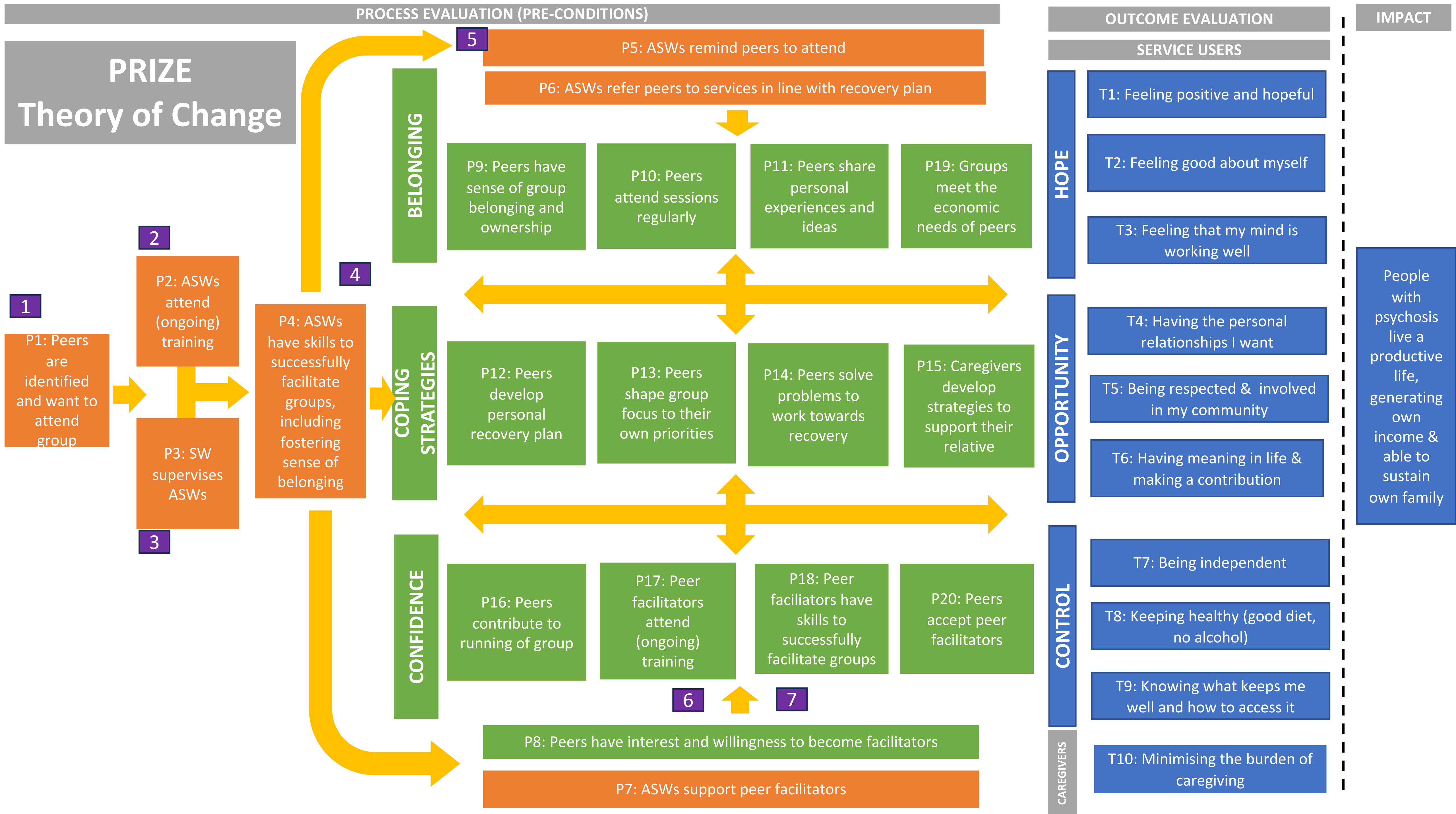
PROCESS EVALUATION (PRE-CONDITIONS)
OUTCOME EVALUATION
IMPACT
PRIZE
Theory of Change
SERVICE USERS
5
P5: ASWs remind peers to attend
P6: ASWs refer peers to services in line with recovery plan
BELONGING
P9: Peers have sense of group belonging and ownership
P10: Peers attend sessions regularly
P11: Peers share personal experiences and ideas
P19: Groups meet the economic needs of peers
HOPE
T1: Feeling positive and hopeful
T2: Feeling good about myself
T3: Feeling that my mind is working well
1
P1: Peers are identified and want to attend group
2
P2: ASWs attend (ongoing) training
3
P3: SW supervises ASWs
4
P4: ASWs have skills to successfully facilitate groups, including fostering sense of belonging
COPING STRATEGIES
P12: Peers develop personal recovery plan
P13: Peers shape group focus to their own priorities
P14: Peers solve problems to work towards recovery
P15: Caregivers develop strategies to support their relative
OPPORTUNITY
T4: Having the personal relationships I want
T5: Being respected & involved in my community
T6: Having meaning in life & making a contribution
People with psychosis live a productive life, generating own income & able to sustain own family
CONFIDENCE
P16: Peers contribute to running of group
P17: Peer facilitators attend (ongoing) training
P18: Peer faciliators have skills to successfully facilitate groups
P20: Peers accept peer facilitators
CONTROL
T7: Being independent
T8: Keeping healthy (good diet, no alcohol)
T9: Knowing what keeps me well and how to access it
6
7
P8: Peers have interest and willingness to become facilitators
P7: ASWs support peer facilitators
CAREGIVERS
T10: Minimising the burden of caregiving

Key Activities

1: ASW makes home visit to participants to explain group purpose and structure

2: Training provided to ASWs in group facilitation

3: Training provided to SW in supervision

4: Weekly groups facilitated by ASWs,

5: Reminder system set up, including register of phone calls and reminder cards

6: Training provided to peer facilitators

7: Weekly groups facilitated by peers

Key assumptions

- Service users attend mental health clinics in sufficient numbers for group formation
- In person group meetings are permitted
- Peers are contactable between group meetings
- Adequate mental health services are available and accessible to peers
