# Supplementary material for: Peer-led recovery groups for people with psychosis in South Africa (PRIZE): Results of a randomized controlled feasibility trial
Source: Epidemiol Psychiatr Sci. 2024 Oct 11;33:e47. doi: 10.1017/S2045796024000556 (PMC11561686; doi:10.1017/S2045796024000556)
Supplement: Asher et al. supplementary material 3 — Asher et al. supplementary material [file S2045796024000556sup003.docx]

**Supplementary file 2**

Table PRIZE quantitative outcome measures and instruments

| **Outcome** | **Measure** | **Details** |
| --- | --- | --- |
| Disability | Self-rated and proxy-rated 12- item WHO Disability Assessment Schedule (WHODAS) 2.0 | The 12-item WHODAS 2·0 is a generic instrument for assessing disability relating to any health condition across cultures. It has been used previously in South Africa for persons with severe mental illness (Brooke-Sumner et al., 2018). Item-response theory-based scoring will be used to convert scores to a 0-100 scale (WHO, 2010). The WHODAS proxy version has the same properties as the self-rated version but has been designed to be answered by a caregiver, relative or friend (WHO, 2010). Number of days in the last month with reduced ability to work and completely unable to work will be assessed using self and proxy report. |
| Recovery | Recovery Assessment Scale- Domains and Stages (RAS- DS) | The RAS-DS is a self-report measure of mental health recovery (Hancock et al., 2014). It includes 38 items clustered into four domains of recovery: functional recovery (“Doing things I value”); personal recovery (“Looking forward”); clinical recovery (“Mastering my illness”); and social recovery (“Connecting and belonging”). Each item is rated on a 4-point scale from 1 = “untrue” to 4 = “completely true”.  “Percentage scores” are calculated for each domain and an overall score Higher scores represent more advanced levels of mental health recovery. It has been used previously in clinical settings in South Africa. |
| Unmet needs | Camberwell Assessment of Need – Short Appraisal Scale (CANSAS) | The CANSAS includes a list of 22 areas considered as potentially important needs for individuals living with mental illness. Each item is rated as either an “unmet need”; “met need” or “no need”. Total number of unmet needs will be presented. The scale has been previously used in South Africa (Brooke-Sumner et al., 2018). |
| Support for recovery | Brief INSPIRE | The brief INSPIRE assesses recovery support from a worker and has 5 items, each rated 0 “not at all” to 4 “very much”. Responses can be converted to total score, ranging from 0 (low recovery support) to 100 (high recovery support) (Williams et al., 2015). |
| Internalised stigma | Internalised stigma of mental illness (ISMI) scale | The ISMI-R is a 29-item questionnaire assessing internalised stigma covering four subscales: ‘alienation’; ‘stereotype endorsement’; ‘perceived discrimination’; and ‘social withdrawal’. Items are scored on a 4-point Likert scale, from strongly disagree to strongly agree. The mean score across all items will be presented. The scale has been previously used in South Africa (Brooke-Sumner et al., 2018). |
| Perception of respect and value | 2 bespoke questions | Two questions ‘I feel valued and respected by my family’ and ‘I feel valued and respected by my community’ will be rated on a 4-point Likert scale, from strongly disagree to strongly agree. The proportion disagreeing or strongly disagreeing to each item will be presented. |
| Alcohol use | Alcohol use disorders identification test consumption (AUDIT C) | The AUDIT-C includes three questions on alcohol consumption, each rated on a 0 to 4 scale. It has been widely used and shown to be useful for assessing alcohol use in the South African context (Morojele et al., 2017). To determine hazardous drinking a cut off of ≥3 (female) or ≥4 (male) will be used. |
| Health service use | Bespoke questions | Visit to mental health nurse in the last 2 months will be assessed. |
| Relapse | Questions on police contact & hospitalization | Relapse is defined as either of:   - Inpatient admission for mental health of any duration (assessed as part of health service use) - Any type of police contact related to mental health |
| Medication adherence | 1 question | We will use a 5-point nominal scale measuring frequency of medication adherence. Non-adherence will be defined as taking medication sometimes, occasionally or never in the last 1 month. |
| Caregiver burden | Caregiving consequences section of Involvement Engagement Questionnaire (IEQ) | 31-item questionnaire assessing aspects of burden for caregivers of persons with severe mental illness. All items are scored on 5-point Likert scales (0 never to 4 always). Domain scores can be computed (tension & urging range 0 to 36; worrying & supervision range 0 to 24). The total score will be used. |

BROOKE-SUMNER, C., SELOHILWE, O., SPHIWE MAZIBUKO, M. & PETERSEN, I. 2018. Process Evaluation of a Pilot Intervention for Psychosocial Rehabilitation for Service Users with Schizophrenia in North West Province, South Africa. *Community Mental Health Journal*.

HANCOCK, N., SCANLAN, J. N., HONEY, A., BUNDY, A. C. & O’SHEA, K. 2014. Recovery Assessment Scale – Domains and Stages (RAS-DS): Its feasibility and outcome measurement capacity. *Australian & New Zealand Journal of Psychiatry,* 49**,** 624-633.

MOROJELE, N. K., NKOSI, S., KEKWALETSWE, C. T., SHUPER, P. A., MANDA, S. O., MYERS, B. & PARRY, C. D. 2017. Utility of Brief Versions of the Alcohol Use Disorders Identification Test (AUDIT) to Identify Excessive Drinking Among Patients in HIV Care in South Africa. *J Stud Alcohol Drugs,* 78**,** 88-96.

WHO 2010. Measuring health and disability: Manual for WHO Disability Assessment Schedule (WHODAS 2.0). Geneva: World Health Organization.

WILLIAMS, J., LEAMY, M., BIRD, V., LE BOUTILLIER, C., NORTON, S., PESOLA, F. & SLADE, M. 2015. Development and evaluation of the INSPIRE measure of staff support for personal recovery. *Soc Psychiatry Psychiatr Epidemiol,* 50**,** 777-86.
