# Supplementary material for: Peer-led recovery groups for people with psychosis in South Africa (PRIZE): Results of a randomized controlled feasibility trial
Source: Epidemiol Psychiatr Sci. 2024 Oct 11;33:e47. doi: 10.1017/S2045796024000556 (PMC11561686; doi:10.1017/S2045796024000556)
Supplement: Asher et al. supplementary material 4 — Asher et al. supplementary material [file S2045796024000556sup004.docx]

**PRIZE 2 month outcome analysis**

|  | **Intervention (TAU + recovery groups) (n=45)** | **Control (TAU)**  **(n=41)** | **Mean difference or risk difference (95% CI)** |
| --- | --- | --- | --- |
| **Disability** |  |  |  |
| **Self-reported total WHODAS (mean [SD])** | 9.38 (11.27) | 8.60 (7.75) | 0.93 (-3.27; 5.13)^a^ |
| **Self-reported days totally unable to work (mean [SD])** | 0.53 (2.34) | 0.22 (1.26) | 0.32 (-0.73; 1.36)^a^ |
| **Self-reported days reduced ability to work (mean [SD])** | 0.33 (1.54) | 0.22 (0.69) | 0.12 (-0.53; 0.77)^a^ |
| **Proxy-reported total WHODAS (mean [SD])** | 7.26 (12.15) | 10.19 (12.75) | -2.61 (-8.03; 2.8)^a^ |
| **Proxy-reported days totally unable to work (mean [SD])** | 2.35 (7.21) | 4.78 (8.66) | -2.47 (-9.91; 4.96)^a^ |
| **Proxy-reported days reduced ability to work (mean [SD])** | 1.12 (2.94) | 1.44 (2.79) | -0.07 (-2.05; 1.91)^a^ |
| **Relapse** |  |  |  |
| **Relapse in last 2 months (endpoint data only) (n [%])** | 1 (2.2%) | 1 (2.4%) | -0.002 (-0.066; 0.062)^a^ |
| **Health service use** |  |  |  |
| **No contact with mental health nurse last 2 months (n [%])** | 0 (0%) | 1 (2%) | 0.024 (-0.023; 0.072)^b^ |
| **Stigma** |  |  |  |
| **Internalized stigma (ISMI) mean score (SD)** | 2.2 (0.5) | 2.1 (0.7) | 0.01 (-0.15; 0.16)^a^ |
| **Does not feel valued and respected by family (n [%])** | 1 (2%) | 2 (5%) | -0.26 (-1.07; 0.55)^a^ |
| **Does not feel valued and respected by community(n [%])** | 3 (7%) | 3 (7%) | 0.14 (-0.42; 0.7)^a^ |
| **Recovery** |  |  |  |
| **Recovery (RAS-DS) total score (mean [SD])** | 80.8 (14.6) | 80.7 (14.8) | 0.04 (-4.88; 4.95)^a^ |
| **Unmet needs** |  |  |  |
| **Number of unmet needs (CANSAS) (mean [SD])** | 1.7 (1.7) | 1.9 (2.0) | -0.16 (-0.64; 0.31)^a^ |
| **Medication adherence** |  |  |  |
| **Non-adherent to antipsychotic medication (n [%])** | 1 ( 2%) | 1 ( 2%) | 0.002 (-0.001;0.005)^a^ |
| **Hazardous drinking** |  |  |  |
| **AUDIT-C total ≥3 (female) or ≥4 (male) (n [%])** | 45 (100%) | 41 (100%) | 0 |
| **Caregiver burden** |  |  |  |
| **Caregiver burden mean IEQ score (SD)** | 13.0 (7.2) | 16.9 (18.3) | -3.27 (-8.49; 1.95) ^a^ |

^a^ Adjusted for baseline score of outcome variable and clinic

^b^ Unadjusted analysis due to low numbers
