# Supplementary material for: Peer-led recovery groups for people with psychosis in South Africa (PRIZE): Results of a randomized controlled feasibility trial
Source: Epidemiol Psychiatr Sci. 2024 Oct 11;33:e47. doi: 10.1017/S2045796024000556 (PMC11561686; doi:10.1017/S2045796024000556)
Supplement: Asher et al. supplementary material 5 — Asher et al. supplementary material [file S2045796024000556sup005.docx]

**PRIZE GroupACT analysis**

|  |  | ASW | |  | | | Peer |  |
| --- | --- | --- | --- | --- | --- | --- | --- | --- |
| Item | Level | Week 1 | | Week 8 | | | Week 1-3 |  |
|  |  | N (%) | Mean (SD) | | N (%) | Mean (SD) | N (%) | Mean (SD) |
| 1. Establish or confirm ground rules | 1: Unhelpful/ harmful behaviour | 0 | 3.5 (1) | | 0 | 4 (0) | 0 | 2.4 (0.7) |
|  | 2: No basic or some but not all basic skills | 1 (25%) |  |  | 0 |  | 11 (79%) |  |
|  | 3: All basic skills | 0 |  |  | 0 |  | 1 (7%) |  |
|  | 4: All basic + any advanced skill | 3 (75%) |  |  | 4 (100%) |  | 2 (14%) |  |
| 1. Group participation | 1: Unhelpful/ harmful behaviour | 0 | 4 (0) | | 0 | 4 (0) | 0 | 2.1 (0.3) |
|  | 2: No basic or some but not all basic skills | 0 |  |  | 0 |  | 12 (92%) |  |
|  | 3: All basic skills | 0 |  |  | 0 |  | 1 (8%) |  |
|  | 4: All basic + any advanced skill | 4 (100%) |  |  | 4 (100%) |  | 0 |  |
| 1. Fostering empathy | 1: Unhelpful/ harmful behaviour | 0 | 4 (0) | | 0 | 4 (0) | 0 | 2.3 (0.7) |
|  | 2: No basic or some but not all basic skills | 0 |  |  | 0 |  | 12 (86%) |  |
|  | 3: All basic skills | 0 |  |  | 0 |  | 0 |  |
|  | 4: All basic + any advanced skill | 4 (100%) |  |  | 4 (100%) |  | 2 (14%) |  |
| 1. Problem solving | 1: Unhelpful/ harmful behaviour | 0 | 2 (0) | | 0 | 4 (0) | 0 | 2.1 (0.5) |
|  | 2: No basic or some but not all basic skills | 4 (100%) |  |  | 0 |  | 13 (93%) |  |
|  | 3: All basic skills | 0 |  |  | 0 |  | 0 |  |
|  | 4: All basic + any advanced skill | 0 |  |  | 4 (100%) |  | 7 (%) |  |
| 1. Barriers to attendance | 1: Unhelpful/ harmful behaviour | 0 | 2 (0) | | 0 | 4 (0) | 13 (93%) | 2.1 (0.5) |
|  | 2: No basic or some but not all basic skills | 4 (100%) |  |  | 0 |  | 0 |  |
|  | 3: All basic skills | 0 |  |  | 0 |  | 1 (7%) |  |
|  | 4: All basic + any advanced skill | 0 |  |  | 4 (100%) |  |  |  |
| 1. Confidentiality | 1: Unhelpful/ harmful behaviour | 0 | 2 (0) | | 0 | 4 (0) | 0 | 2 (0) |
|  | 2: No basic or some but not all basic skills | 4 (100%) |  |  | 0 |  | 14 (100%) |  |
|  | 3: All basic skills | 0 |  |  | 0 |  | 0 |  |
|  | 4: All basic + any advanced skill | 0 |  |  | 4 (100%) |  | 0 |  |
| 1. Time management | 1: Unhelpful/ harmful behaviour | 0 | 2.5 (1) | | 0 | 4 (0) | 0 | 2 (0) |
|  | 2: No basic or some but not all basic skills | 3 (75%) |  |  | 0 |  | 14 (100%) |  |
|  | 3: All basic skills | 0 |  |  | 0 |  |  |  |
|  | 4: All basic + any advanced skill | 1 (25%) |  |  | 4 (100%) |  |  |  |
| Total |  |  | 2.9 (2.3) | |  | 4 (0) |  | 2.1 (0.3) |
