# Supplementary material for: Peer-led recovery groups for people with psychosis in South Africa (PRIZE): Results of a randomized controlled feasibility trial
Source: Epidemiol Psychiatr Sci. 2024 Oct 11;33:e47. doi: 10.1017/S2045796024000556 (PMC11561686; doi:10.1017/S2045796024000556)
Supplement: Asher et al. supplementary material 6 — Asher et al. supplementary material [file S2045796024000556sup006.docx]

**Baseline characteristics of completers of outcome evaluation and those lost to follow up at 5 months**

|  | **Lost before 5 month evaluation** | **Completed 5 month evaluation** | **P value** |
| --- | --- | --- | --- |
| n | **11** | **81** |  |
| **Sex (n [%])** |  |  |  |
| Male | 9 (82%) | 59 (73%) | 0.52 |
| Female | 2 (18%) | 22 (27%) |  |
| **Age (years)** **(mean [SD])** | 41.5 (11.9) | 46.2 (11.5) | 0.20 |
| **Race (n [%])** |  |  |  |
| African | 11 (100%) | 81 (100%) | - |
| **Marital status (n [%])** |  |  |  |
| Married | 0 (0%) | 4 ( 5%) | 0.64 |
| Widow/widower | 0 (0%) | 4 ( 5%) |  |
| Divorced or separated | 0 (0%) | 3 ( 4%) |  |
| Never married (single) | 11 (100%) | 70 (86%) |  |
| **Employment status** **(n [%])** |  |  |  |
| Unemployed and looking for work | 6 (55%) | 29 (36%) | 0.57 |
| Unemployed and not looking for work | 5 (45%) | 45 (56%) |  |
| Employed part-time | 0 (0%) | 5 (6%) |  |
| Pensioner | 0 (0%) | 2 (2%) |  |
| **Education status (n [%])** |  |  |  |
| Primary education | 3 (27%) | 28 (35%) | 0.11 |
| Secondary education | 5 (45%) | 47 (58%) |  |
| Diploma/degree | 3 (27%) | 6 (7%) |  |
| **Problems with learning** **(n [%])** |  |  |  |
| No | 8 (73%) | 64 (79%) | 0.64 |
| Yes | 3 (27%) | 17 (21%) |  |
| **Living situation** **(n [%])** |  |  |  |
| I have a place to live where I can stay as long as I want | 11 (100%) | 79 (98%) | 0.60 |
| I currently have a place to live, but may not be able to stay there in the future | 0 (0%) | 2 (2%) |  |
| **Main source of income (n [%])** |  |  |  |
| Odd jobs | 0 ( 0%) | 7 (9%) | 0.20 |
| Government grant (childhood /disability) | 8 (73%) | 66 (81%) |  |
| No income | 2 (18%) | 7 (9%) |  |
| Other | 1 ( 9%) | 1 (1%) |  |
| **Monthly income (n [%])** |  |  |  |
| Less than R600 (32 USD) | 2 (18%) | 14 (17%) | 0.85 |
| R600-1000 (32- 53 USD) | 0 ( 0%) | 1 ( 1%) |  |
| R1001-2000 (54- 107 USD) | 9 (82%) | 58 (72%) |  |
| R2001-4000 (108- 213 USD) | 0 (0%) | 7 ( 9%) |  |
| Dont know | 0 (0%) | 1 ( 1%) |  |
| **Self-reported total WHODAS (mean [SD]*)** | 5.8 (6.4) | 8.3 (10.2) | 0.43 |
| **Relapse in last 2 months (n [%])** |  |  |  |
| No | 9 (82%) | 78 (96%) | 0.107 |
| Yes | 2 (18%) | 3 ( 4%) |  |
| **Internalized stigma total score (mean [SD])** | 2.5 (0.8) | 2.4 (0.7) | 0.74 |
| **Recovery (RAS-DS) total score (mean [SD])** | 92.27 (18.23) | 89.04 (17.11) | 0.51 |
| **Number of unmet needs (CANSAS) (mean [SD])** | 1.64 (1.50) | 1.53 (1.44) | 0.82 |
| **Percentage of unmet needs (CANSAS) (mean [SD])*** | 25.4 (6.1) | 39.4 (3.7) | 0.1722 |
| **Contact with mental health nurse last 2 months (n [%])** |  |  |  |
| No | 0 ( 0%) | 2 ( 2%) | 0.60 |
| Yes | 11 (100%) | 79 (98%) |  |
| **Antipsychotic medication adherence (n [%])** |  |  |  |
| All the time | 10 (91%) | 81 (100%) | 0.006 |
| Most of the time (> 3 of the last 4 weeks) | 1 ( 9%) | 0 ( 0%) |  |
| Sometimes, occasionally, or not at all | 0 | 0 |  |
| **AUDIT-C total ≥3 (female) or ≥4 (male) (n [%])** |  |  |  |
| No | 8 (73%) | 67 (83%) | 0.42 |
| Yes | 3 (27%) | 14 (17%) |  |
| **Caregiver burden mean IEQ score (SD)** | 13.25 (3.03) | 17.24( 1.82) | 0.512 |
