# Supplementary material for: Peer-led recovery groups for people with psychosis in South Africa (PRIZE): Results of a randomized controlled feasibility trial
Source: Epidemiol Psychiatr Sci. 2024 Oct 11;33:e47. doi: 10.1017/S2045796024000556 (PMC11561686; doi:10.1017/S2045796024000556)
Supplement: Asher et al. supplementary material 7 — Asher et al. supplementary material [file S2045796024000556sup007.docx]

**Supplementary File 7 5 month analysis by caregiver status**

|  | **Treatment as usual**  **(n=39)** | **Recovery groups and treatment as usual (n=42)** | **Mean difference or risk difference (95% CI)** |
| --- | --- | --- | --- |
| **Disability** |  |  |  |
| **Self-reported total WHODAS (mean [SD])** |  |  |  |
| **Service users with caregiver** | 6.4 (3.9) | 7.6 (12.0) | 1.18 (-4.92, 7.29) |
| **Service users without caregiver** | 5.4 (4.7) | 6.9 (5.5) | 1.43 (-1.88, 4.75) |
| **Self-reported days totally unable to work (mean [SD])** |  |  |  |
| **Service users with caregiver** | 0 (0) | 0.6 (3.0) | 0.06 (-0.88, 2.08) |
| **Service users without caregiver** | 0.1 (0.4) | 0 (0) | -0.09 (-0.30, 0.12) |
| **Self-reported days reduced ability to work (mean [SD])** |  |  |  |
| **Service users with caregiver** | 0 (0) | 0.4 (2.0) | 0.40 (-0.58, 1.38) |
| **Service users without caregiver** | 0.5 (2.1) | 0.3 (1.2) | -0.16 (-1.33, 1.01) |
| **Relapse** |  |  |  |
| **Hospitalisation or police contact in last 2 months (interview and SAE data) (n [%]) (n=92)** |  |  |  |
| **Service users with caregiver** | 3 (15.8%) | 1 (3.7%) | -0.12 (-0.03, 0.06) |
| **Service users without caregiver** | 5 (18.5%) | 0 (0%) | -0.19 (-0.33, -0.04) |
| **Health service use** |  |  |  |
| **No contact with mental health nurse last 2 months (n [%])** |  |  |  |
| **Service users with caregiver** | 0 (0) | 1 (4.0%) | - |
| **Service users without caregiver** | 0 (0) | 0 (0) | - |
| **Stigma** |  |  |  |
| **Internalized stigma (ISMI) mean score (SD)** |  |  |  |
| **Service users with caregiver** | 1.9 (0.6) | 1.9 (0.5) | 0.05 (-0.39, 0.29) |
| **Service users without caregiver** | 1.8 (0.6) | 2.0 (0.6) | 0.16 (-0.23, 0.55) |
| **Does not feel valued and respected by family (n [%])** |  |  |  |
| **Service users with caregiver** | 0 (0) | 1 (4.0%) | 0.04 (-0.04, 0.12) |
| **Service users without caregiver** | 1 (4.6%) | 2 (11.8%) | 0.07 (-0.10, 0.25) |
| **Does not feel valued and respected by community (n [%])** |  |  |  |
| **Service users with caregiver** | 3 (17.6%) | 1 (4.0%) | -0.14 (0.33, 0.06) |
| **Service users without caregiver** | 1 (2.6%) | 3 (17.7%) | 0.13 (-0.07, -0.33) |
| **Recovery** |  |  |  |
| **RAS-DS total score (mean [SD])** |  |  |  |
| **Service users with caregiver** | 84.6 (7.5) | 88.5 (9.3) | 3.95 (-1.54, 9.5) |
| **Service users without caregiver** | 85.1 (12.8) | 81.3 (12.7) | -3.77 (-12.10, 4.57) |
| **Unmet needs** |  |  |  |
| **Number of unmet needs (CANSAS) (mean [SD])** |  |  |  |
| **Service users with caregiver** | 1.6 (1.2) | 1.6 (1.2) | 0.01 (-0.73, 0.75) |
| **Service users without caregiver** | 1.5 (1.5) | 1.4 (1.5) | -0.15 (-1.12, 0.82) |
| **Medication adherence** |  |  |  |
| **Non-adherent to antipsychotic medication (n [%])** |  |  |  |
| **Service users with caregiver** | 0 (0) | 0 (0) | - |
| **Service users without caregiver** | 0 (0) | 1 (5.9%) | - |
| **Hazardous drinking** |  |  |  |
| **AUDIT-C total ≥3 (female) or ≥4 (male) (n [%])** |  |  |  |
| **Service users with caregiver** | 4 (23.5%) | 7 (28.0%) | 0.04 (-0.22, 0.31) |
| **Service users without caregiver** | 3 (13.6%) | 1 (5.9%) | -0.08 (-0.26, 0.10) |
